# The gift remembered: The Human Anatomy and Physiology Society (HAPS) Anatomical Donor Stewardship's ethical and practical handbook for anatomy donor tributes

**DOI:** 10.1002/ase.70309

**Published:** 2026-07-16

**Authors:** Bobbie J. Leeper, Rhiannon E. Robinson, Kelsey Stevens, Lacy M. Cleveland, Danielle N. Edwards, Nicole L. Geske, Burhan Gharaibeh, Anya Goldina, Jill Kirby, Jacqueline Phillips, Leslie R. Sewell, J. P. Swigart, Trisha Waldman, Jonathan J. Wisco, Jeremy J. Grachan

**Affiliations:** ^1^ Biology and Physician Assistant Programs Seton Hill University Greensburg Pennsylvania USA; ^2^ Department of Anatomy & Neurobiology Boston University Chobanian & Avedisian School of Medicine Boston Massachusetts USA; ^3^ Department of Exercise and Health Sciences Briar Cliff University Sioux City Iowa USA; ^4^ School of Science and Engineering Colorado Christian University Lakewood Colorado USA; ^5^ Department of Anatomical Science and Neurobiology University of Louisville School of Medicine Louisville Kentucky USA; ^6^ Radiology Michigan State University East Lansing Michigan USA; ^7^ Department of Biological Sciences University of Pittsburgh Pittsburgh Pennsylvania USA; ^8^ Department of Biological and Environmental Sciences Elizabethtown College Elizabethtown Pennsylvania USA; ^9^ Department of Biology Lipscomb University Nashville Tennessee USA; ^10^ Department of Health Sciences Drexel University Philadelphia Pennsylvania USA; ^11^ Division of Anatomy The Ohio State University College of Medicine Columbus Ohio USA; ^12^ Biomedical and Translational Sciences Carle Illinois College of Medicine Urbana Illinois USA; ^13^ School of Arts and Sciences University of Mary Bismarck North Dakota USA; ^14^ Department of Medicine Rutgers New Jersey Medical School Newark New Jersey USA

**Keywords:** anatomy education, body donors, cadavers, ceremony, dissection, ethics, memorials

## Abstract

Providing students with a way to honor human body donors during or after an anatomy course has been shown to help provide a sense of closure and comfort to students and, when present, donor families. Gestures of gratitude for donors can also emphasize humanistic values of respect, empathy, and professionalism. These tributes can take the form of a ceremony; class activities such as a moment of silence or reflection assignment; the display of a memorial plaque, website, plant, or garden; or other similar gestures. While hosting memorial ceremonies for donors has become common practice for many body donation programs and larger medical institutions, many institutions still do not hold ceremonies because faculty do not know where to start, are deterred by the increased workload, or for other personal or administrative reasons. This handbook was created by members of the Human Anatomy and Physiology Society (HAPS) Anatomical Donor Stewardship (ADS) Ethics Subcommittee to provide guidance to anatomy educators and staff who are developing inaugural ceremonies or memorials, or who are seeking new ideas to enhance their established event or memorial.

## INTRODUCTION

Human body donors have been, and continue to be, an essential part of anatomy education for many students in academic and health care programs around the world.^1^, ^2^ It is widely recognized that while other learning modalities may confer the same rote lists of material for students to learn, the intimacy and perspective that human dissection offers helps to develop a student with not just hands‐on experience with human anatomy, but also, importantly, a more well‐rounded and empathetic healthcare professional.^3^, ^4^ When surveyed, students feel that dissection is an irreplaceable experience that helps them not only learn anatomy, but also recall theoretical knowledge^5^ and develop practical surgical skills.^6^ So impactful is this relationship that the terms “silent mentors” and “silent teachers” have been used by some anatomists in recent years to recognize the importance of donor bodies in the laboratory.^7^ Nevertheless, a donor's gift does not come without sacrifice.

Human anatomy dissection is made possible through the selfless gift of body donation. In the United States, full body donation is a separate and often more involved process than registering for organ donation, which usually only involves checking a box on a driver's license form or medical form.^8^ Whole‐body donation is commonly made in either of two ways: self‐donation or next‐of‐kin donation.^9^ Whole body donation, whether self or next‐of‐kin, requires registration through a state or institutional body donation program, requires active notification (e.g., telling next of kin to ensure this is carried out, putting a card in your wallet, etc.), and possibly requires an addition to the donor's will.^10^ Therefore, most anatomical body donors will have had the foresight and initiative to reach out while of sound mind and body, to make arrangements with the donation organization, to incorporate that information into important end‐of‐life documents, and to inform their families of their choice. Their gift of donation is not only a gift during death, but also one of significant time commitments during their life. However, not all bodies utilized in anatomy laboratories are bequeathed, as the bodies of unclaimed individuals are still utilized worldwide.^11^ Although these individuals do not arrive at the anatomy laboratory through their own altruistic decision, since consent was not obtained by them or their family, their post‐mortem sacrifice should be honored just as those who donated their bodies.

Finally, the treatment of anatomical donor remains can be saddled with unsavory perceptions by the general public. Some people inherently distrust the medical establishment due to a long history of abuse towards marginalized populations (e.g., black Americans and medical experimentation during the mid‐20th century), the history of grave‐robbers hired by medical schools, or based on improprieties in the news from more recent times.^12^, ^13^ These feelings are valid and need to be respected. As stewards of anatomical donors, anatomists bear the burden of responsible and moral treatment of the dead within the academic setting, and owe it to the donors, their families, and the general public to handle donors with the highest ethical and practical standards.^13^, ^14^ Donors should be treated with at least as much reverence as they would receive in other funerary settings. Respectful treatment honors the donor's gift, empathizes with the family's grief, and can help reassure a wary public that we are to be trusted.

One way in which we demonstrate gratitude for the donors' gift of their bodies is by hosting memorial ceremonies. Memorial ceremonies draw from basic humanistic practices that have been undertaken since our most ancient cultural beginnings and resonate with us in powerful ways.^15^ While similar to public memorial ceremonies, the memorial ceremony in anatomy education fosters respect and gratitude for the donors from those who typically knew them only in death, not life.^14^, ^16^, ^17^, ^18^, ^19^, ^20^, ^21^, ^22^ Additionally, a formal ceremony provides a meaningful opportunity for students and their instructors to engage in a project that emphasizes the human aspect of their studies as well as a means for closure to the dissection experience and a means to cope with the complex emotions that accompany that conclusion.^14^, ^17^, ^22^ A ceremony can also provide a means to highlight feelings of respect and gratitude for remains of non‐donated individuals or of unknown origins, including unclaimed bodies or legacy collections of fetal remains, long‐term prosections, or skeletal remains.^23^ Successful planning and execution require collaboration, empathy, and respect for all involved.

The purpose of this handbook is to provide support and guidance for those who wish to hold a memorial or appreciation ceremony to honor any and all human remains used in anatomy courses, which can include remains of body donors, unclaimed individuals, or of unknown provenance from legacy collections. This handbook was crafted by the Human Anatomy and Physiology Society (HAPS) Anatomical Donor Stewardship (ADS) subcommittee on Ethics. ADS committee members' experience has shown that hosting some form of honoring ceremony, while maintaining best practices in the laboratory, is important in creating an environment that is respectful to the donors. These practices emphasize the appreciation for the donation, support the humanistic values of respect and dignity that accompany human dissections when approached with the new ethical ethos of anatomy education,^24^ and may provide a sense of closure and comfort for those involved. While many institutions host some form of honoring practice, others may not out of concerns about the workload, hesitations from administration, or uncertainty of where to begin.

There has been a significant number of publications detailing specific memorial ceremonies and other tribute activities across the world, including Australia and New Zealand,^25^, ^26^, ^27^ Brazil,^28^, ^29^ Europe,^20^, ^30^, ^31^ India,^16^, ^32^, ^33^, ^34^, ^35^ South Korea,^36^, ^37^ Taiwan,^38^, ^39^, ^40^, ^41^, ^42^ and the United States,^17^, ^18^, ^22^, ^43^, ^44^, ^45^, ^46^, ^47^, ^48^, ^49^, ^50^, ^51^, ^52^ as well as an entire book dedicated to the topic of donor memorials and ceremonies^21^; however, no publication has offered a curated list of suggestions and considerations for how to organize and implement a ceremony. This handbook supports educators as they work to create or modify their ceremonies or practices by providing resources and experiences from all those involved in its authorship. The reader should bear in mind that this handbook is a list of suggestions and advice, assembled and curated by fellow educators and anatomists; it is not meant to be comprehensive or prescriptive. There is no “correct” way to hold a ceremony, aside from the one that best suits the donors, their families, the students, and the institution.

### Notes on terminology

This handbook utilizes the term “human body donor,” “anatomical donor,” or simply “donor” to refer to the bodies in anatomy laboratories used for dissection or prosection. Any form of the term “donor” reflects the person's humanity and their (or their family's) decision to donate their body. Therefore, “donor” is preferred over terms like “cadaver” or “body” that objectify the donors.^14^, ^35^, ^52^, ^53^, ^54^, ^55^ This handbook also frequently employs the word “institution”; however, all suggestions made in this handbook can be applied at the level of the classroom, the department, or the whole school/university.

### Purpose of a ceremony or other honoring practices

While the practices of a funeral and a memorial ceremony may appear outwardly similar, the intent behind each differs. A funeral ceremony's purpose, execution, and meaning may be driven by cultural, religious, or psychological needs. Funerals honor the life of the decedent and allow those who were connected to them an opportunity to mourn, respect, receive closure, pray, offer gifts for the afterlife, or mark the end of a shared community.

Memorial ceremonies, on the other hand, honor the educational role that the decedent played in their death. Even though students likely did not know the body donors while they were living, they can develop powerful emotions and strong bonds with them throughout a course.^56^ The emotions experienced can be complex and fluidly change throughout the dissection process since students are also encouraged to approach the human anatomy donor in a holistic, humanistic way, remembering that this person is not a lone specimen on a table but a member of a community and of a family. Students can experience feelings of stress, guilt, discomfort, insufficiency, empathy, respect, etc.^56^, ^57^ These complex emotions need to be acknowledged, and students' psychological needs for safety and comfort must be attended to during the duration of a gross anatomy course featuring an anatomical donor.^58^ Surveys of student perceptions following donor memorial ceremonies show the clear benefits, particularly in easing feelings of discomfort or stress associated with the dissection process,^59^ fostering empathy which can assist in developing better relationships with patients,^29^ and providing a sense of closure.^17^

Ceremonies can also provide a space for donor families to express grief and gain a sense of closure after the death of their loved one. Families of the decedent are left as indirect participants in the anatomical donation process, which can further complicate their own grief.^17^, ^60^ Grief and closure in this context deserve the same acknowledgment and respect as those relationships kindled during life.

Memorial ceremonies and other tribute practices demonstrate respect and appreciation for the donor and the donor's family, and can be in the form of small gestures throughout the course (e.g., washing the donor, moments of silence, reflection activities, etc.) as well as a formal ceremony. These tributes can also provide closure to students and their own emotional journeys throughout and after the course.^17^, ^22^, ^50^ The gift of the donor's body is emphasized, and gratitude for the generosity and patience of the donor's family is outwardly expressed. The humanity of the donor is acknowledged, and as such, the lens through which students will approach other patients (living or deceased) during their future careers is created.

### Types of tribute practices

The terms *ceremony* and *memorial* may sound synonymous, but each term refers to different means by which one can honor anatomical donors.^21^ A *ceremony* is an event in which participating people gather and participate in an organized, ritualized event focusing on, in this context, the anatomical donor and the gift of their body for students' education. Other terms often used in place of *ceremony* include *memorial service*, *remembrance*, or *observance*. This event can be as simple or elaborate as the organizers want to make it, as the meaning behind the ceremony can shine through regardless of the setting. In contrast, a *memorial* is a physical, permanent marker noting for posterity the contribution of a particular anatomical donor or donors. Memorials can take the form of a shared gravesite for their cremated remains with a traditional upright granite monument, a plaque outside of the anatomy laboratory commemorating the donors, a tree planted in their honor, a statement of acknowledgment featured prominently on the department website, and so forth. A *memorial* used in the singular is a physical item or place and should not be confused with a *memorial ceremony* or *memorialization*, both of which refer to a ceremonial event.

A *token of remembrance* is a physical gift that is offered to the donor or the donor's family directly as a sign of reverence or respect. Examples of tokens of remembrance are rosary beads, flowers, artwork, written notes, etc. If offered to the donor, these items may be removed after the fact or might be interred or cremated with the donor upon final disposition if permitted by the body donation program. A *memorial* is distinct from *tokens of remembrance* in that tokens are not permanent fixtures.

## CONSIDERATIONS IN ORGANIZING A CEREMONY

### First time vs. ongoing planning

Planning a ceremony for the first time will take more time and consideration than planning a subsequent ceremony. For instance, if you are planning your institution's first ceremony, then you will likely have to seek approval for many of the components listed in this handbook. However, do not let this additional work sway you from holding a ceremony since the initial time that you invest in planning will pay off later. Once the initial plan is in place, it can be used as a guide or framework for succeeding ceremonies. Minor changes to the details, such as the music, art, readings, or flowers, can still make a ceremony personalized and meaningful for each class of students. The basic plans, such as the location or any ceremony component that is particularly important for your institution or program, can be made into a checklist that can be used annually to ease the planning process.

Remember that whether you are starting a tribute practice in your department from scratch or you are inheriting a memorial ceremony to organize yourself, you do not have to reinvent the wheel; use past iterations of these ceremonies as a guideline. Reach out to other anatomy departments that are similar in size and scope and find out what they do. You may also utilize the HAPS's ADS subcommittee members as resources for tribute planning (https://www.hapsweb.org/anatomical‐donor‐stewardship‐committee/).

### Ceremony complexity and size

Ceremonies can be elaborate or simple, large or small, public or private, all depending on budget, institutional support, and the needs of the specific program. The scale of the ceremony will depend on the institution's size, if the donor program is housed there, the number of programs that use the donors, and if the donors' families will be present. The type of space that you have available for a ceremony or reception following the ceremony is also a key consideration. Some institutions utilize a larger conference room or classroom, chapels, auditoriums, or even the atrium of a larger campus building for ceremonies and receptions. Outdoor spaces or an off‐campus secondary location may also be appropriate depending on the number of participants, administrative support, availability, weather, event needs, funds, and religious or cultural tone.

The act of honoring donors holds value for all students involved in human dissection since it has been shown that ceremonies provide a means for closure as well as an additional method to foster empathy, respect, and professionalism.^17^, ^22^, ^28^, ^50^ Therefore, keep all student cohorts, including anatomy and anthropology as well as medical or allied health students, in mind when estimating attendance for your ceremony. It may also be prudent to include the other faculty members who work with the involved cohorts.

Ceremonies meant to honor an institution's donors may already be held by the larger‐entity donation program, but these ceremonies do not prevent or limit your institution from hosting a ceremony to honor the donors you take into your care. Students at your institution might be invited to the larger donor institution ceremony; however, by providing your own ceremony, students have the opportunity to reflect and honor the donors with whom they have worked. Furthermore, a more intimate ceremony with your students separate from a larger unknown group may be helpful, as these students have a specific shared experience. If a large formal ceremony is not possible due to time or budget constraints, a simple act of providing students time for a moment of silence and a rose to place on the donor prior to leaving their last laboratory session could be meaningful to the student and also fulfill the goal of providing them with a sense of closure.^61^ Finally, you might consider reaching out to your donor program and asking if there is another way that your students can contribute to their in‐house ceremony, for example, writing thank‐you cards to the donor families or speaking/performing at the ceremony.

When deciding on the scope and complexity of your ceremony, consider the focus of the ceremony. If the focus of the ceremony is to provide a memorial service for the donors' families and show them the impact of their family member's donation, then you should consider finding a large enough location for the event to accommodate the family members and students. A ceremony with this focus is typically more complex, including multiple speakers, music, or selected readings. If the focus of the ceremony is on providing a means for your students to say “thank you” and “goodbye” to the donors, then you could select a simpler form of ceremony, such as just saying a few words and providing a moment of silence in the laboratory with only the students in attendance. However, components of both focus types could be incorporated into a single ceremony so that closure for both students and family members can be addressed and highlighted.

### Organizing a ceremony planning committee

#### Planning committee membership and tasks

Planning a ceremony should not be shouldered by an individual faculty member, administrator, or student due to the work involved and limitations of varying perspectives that could enhance the ceremony components. For larger events, planning committees allow for the division of labor and the utilization of various expertise. Organizing a planning committee can ease the workload and stress involved in planning by dividing the tasks among the committee members. The composition of the planning committee will be determined by how you or your institution wants the organization of the event to be led. Ceremony planning can be driven by either faculty/administration or by students. Regardless of who leads the planning, the planning committee can be made up of members from various roles within the institution. Members may include the donor laboratory administrator(s), administrative representative(s) from the institution (e.g., college event coordinator, program director, or dean), faculty member(s), religious representative(s), and students.

Common elements that all parties should consider when developing their ceremony include program structure, tribute activities, donor and family acknowledgment, and closing comments. The program could start with opening remarks and welcome speeches, followed by reflections from students, faculty, and possibly family members. Tribute activities can include candle lighting, musical performances, and visual tributes such as student‐created artwork. Acknowledging donors and their families can involve letters or tokens of appreciation and inviting donor family members to share their experiences. The ceremony may conclude with final reflections and providing resources for emotional support. For a large ceremony, a planning committee may delegate tasks including:

- Sending invitations and tracking RSVPs.
- Securing an event location.
- Securing parking for guests.
- Collecting pictures from the donor family and developing a slideshow tribute.
- Ordering flowers, candles, etc.
- Program design.
- Securing speakers and performers.

Bear in mind that if you are planning the institution's first memorial ceremony or if you are planning any major changes to an established ceremony, you should consult with administration for approval prior to planning. Administration should especially be consulted when deciding the major aspects of the ceremony such as the size of the event (who all will be involved and invited), the amount or type of religious inclusion, and the location of the event. Your institution may be willing to give you a wide berth in planning, or may want to be intimately involved with the details of the ceremony. Your institution may have concerns about liability (e.g., fire code, liability for non‐student visitors, etc.) and may have strict boundaries surrounding large events. They may also have feelings or guidelines about advertising or publicizing your memorial ceremony (i.e., how or if it can be done). A detailed list of suggested tasks for the planning and implementation of a memorial ceremony is located in the Appendix S1.

#### Student‐led planning

A student‐led approach involves students as the primary planners and executors of the memorial ceremony, with faculty providing support and guidance, especially with the institutional planning process (e.g., room reservation, catering ceremonies, etc.). This approach benefits students by fostering empathy and respect and encouraging active participation in a meaningful project. Having students involved in planning also helps to grow their administrative skills and may help them complete a required component of their program. Students from various programs that utilize and learn from the donors can be invited to volunteer for the committee to add diverse input into planning details as well as students who have assisted in planning ceremonies from previous years.

Having a student‐led ceremony allows the students to provide a more personal ceremony for their silent teachers that reflects the different cultures and customs of the student body. Depending on the size of the courses involved, all students can help or the planning can be left to a committee of representative students. Students should not feel they have to work alone or without guidance. Faculty and administration can help provide a historical framework for the ceremony with examples of past budgets, materials, room reservations, and itineraries. By providing past examples, students have a framework to begin adding their personal touches and it may make the task of planning a ceremony less daunting and stressful for them.

#### Faculty/administration‐led planning

A faculty/administration‐led organization ensures institutional oversight and consistency. Faculty and/or administration take the lead in planning, but students can still provide input and participate in the event. Having a faculty member or administrator organize ceremonies can be more efficient in subsequent iterations since most of the planning has been done and materials can be updated as opposed to wholly created anew. This approach is particularly useful for student populations that may not be able to devote time and effort to planning an extracurricular activity. However, a faculty/administration‐led organized ceremony may seem less meaningful to students unless the faculty take care to express the importance of the ceremony or garner some student input to incorporate.

Forming a planning committee can make organizing the memorial ceremony more manageable so that tasks can be delegated to the members. The composition of the committee can vary but may include a diverse group of individuals, such as student representatives, anatomy and ethics professors, administrative staff, a chaplain or spiritual advisor, body donor organizations, registered body donors, and donor family members. Involving a wide range of participants ensures a comprehensive and respectful approach to planning the ceremony. The committee should be mindful that it is representing the views of the entire student class and the donors to the best of the committee's ability. Therefore, if your institution utilizes this approach to ceremony planning, a major consideration during the planning should be how and if the students' and the donor's family's emotional needs are being met.

### Religious involvement

Religion or faith can be a strong element to help with coping with difficult emotions.^62^ Therefore, the inclusion of religion into a memorial ceremony can be comforting for some attendees (students, faculty, and donor families, etc.) and may fit the affiliation of the university; however, not everyone, including the body donors, shares the same beliefs and faith backgrounds. Therefore, if religion is to be included in the ceremony, whether it be the primary focus or a small component, plans should be made to involve a diverse group of planners with the goal of religious and cultural inclusivity for the ceremony.

Certain religious components such as passages or iconography (e.g., depiction of a cross) on the invitation and/or program or religious prayers, readings, or music in the ceremony may be exclusionary to those who do not practice the same faith. However, you should not avoid faith/religion altogether when planning a ceremony since many students find comfort in their spirituality, which can be used as a coping strategy for dealing with the complex emotions elicited during dissection or the conclusion of a dissection course.^26^, ^27^, ^62^ Including these religious components can be done as long as other faiths are considered and acknowledged so that they are inclusive. Hosting an inter‐faith ceremony that incorporates and embraces multiple faiths can easily be done by representing other religions in all aspects of the ceremony.^47^ For example, passages from various religions can be read, music from multiple faiths can be played, or a final moment of silence could be offered rather than a faith‐specific prayer or blessing.

Ultimately, the way you approach the ceremony will be different depending on your institution and its mission/values. In fact, revisiting your institution's mission statement may provide guidance in determining the right tone or choosing overarching themes for your service. However, consider that everyone is bringing their own reflection and journey to the ceremony; providing space for that diversity is necessary.

### Components to consider during organization

#### Name of event

Determining the name or title of the ceremony should be a consideration that involves university administration, faculty, and students to make sure that it fits with the mission of the university and the goals of the event. The name could be a set title that is used every year, or it could be changed annually and voted on by the students to be more meaningful to them. When selecting a title of the event, note that the term “service” tends to have a religious connotation.^63^ Some example names include the following^21^, ^47^, ^50^:

- Ceremony/Service of Gratitude.
- Donor Memorial Ceremony/Service.
- Remembrance Ceremony/Service
- Ceremony/Service of Thanksgiving.
- Convocation of Thanks.
- Commemoration

If a religious service, such as a mass, is part of your ceremony, then that could be included in the title of the event to highlight the religious focus. However, you should consider inclusivity of other faiths (of the faculty, students, and body donors) since not everyone may follow or be comfortable with a particular religion, even if it is the university's religious affiliation. Some example names that contain “mass” in the title include the following^47^:

- Donor Mass
- Mass of Human Gifts

Another variation to the name or title of the ceremony is to also indicate who is holding the event and the year in which the ceremony is conducted. For example (with [X] representing the institution or program name and year):

- [X] Body Donation Program Memorial Ceremony
- [X] Anatomy Donor Memorial Ceremony
- [X] Donor Memorial Ceremony

#### Timing of the ceremony

Ceremonies may be held annually, at the beginning of a class or semester, at the end of a class/semester/block, or at the culmination of all classes that use the donors. Though ceremonies held prior to beginning dissection are not as common in the United States, they can function to help set a respectful tone in the laboratory once dissections begin since the donors' humanity is emphasized early on.^14^ Chiou and colleagues surveyed students after an initiation ceremony held for donors prior to dissection in Taiwan and found that the event reduced feelings of unease about dissection.^59^ However, a ceremony held at the conclusion of a dissection course can be more meaningful to the students and help provide them with closure to help them deal with emotions evoked during dissections.^17^, ^22^, ^52^ Ceremonies may be held annually, or after some other point of natural closure with those donors. Whether one chooses to hold a ceremony prior to or after learning from the donor, ongoing practices should be implemented throughout the course to encourage respectful treatment of the donors.

Ceremonies at the beginning of the term focus on setting the tone for showing respect, expressing gratitude, and maximizing the learning experience. These ceremonies may be as simple as a blessing provided by clergy, a moment of silence, and/or the presence of counseling staff to assist students in their emotional processing. However, they can be formal ceremonies that involve meeting the donors' families, as practiced as part of the Silent Mentor Program (SMP) in Taiwan.^38^, ^39^, ^40^, ^41^, ^42^ The SMP initiation ceremony has been shown to assist in maintaining the donor's humanity during dissections.^39^

Ceremonies held at the end of the course or year focus on closure for the students and, if invited, donor families. Such ceremonies allow for a final expression of thanks to the donors for their gift. daRocha and colleagues found that students who attended the final ceremony had a more positive view of the body donation process and believed that the ceremony had a positive impact on them developing better doctor‐patient relationships.^29^ Common elements for end of the year/course ceremonies include a welcome, reflections from faculty and students, a tribute (e.g., candlelight vigil, presentation of flowers, performances, etc.), and closing remarks.

Also consider when deciding on a particular date of the ceremony that your academic calendar may not be identical to the calendar of your other participants. For example, students have different needs during finals week than faculty; administration may be busy with admissions during a time that feels quiet for everyone else, breaks may be different between undergraduate and graduate departments, and holidays may be a slow time for the school but a busy time for families. Commuter campuses may need to hold their ceremony at a different time than campuses with a larger on‐campus community. Additionally, consider if a major holiday may affect your ceremony. For example, if the location of your event will be decorated for Christmas, will it detract from the purpose or tone of the ceremony?

#### People involved in the ceremony

##### Donors as the honorees

Though participants' emotions drive many of the decisions when organizing a memorial ceremony, it is vital to remember that individuals who donated their body for the students' education are the ones being honored. As such, it is important to remember all donors involved with your program, including the whole‐body donors who are typically memorialized as well as the regional or single‐structure donations (e.g., limbs, individual organs, etc.) that may be utilized. Brains used in a neuroanatomy course, or other individual structures for other anatomy courses, may not include identifying information like whole‐body tissue, but still represent the selfless contributions of an individual. Legacy collections or bone collections are also commonly overlooked for memorial ceremonies but still represent valuable contributions that can be memorialized to remind students of the sacrifice involved in all human tissue donation or acquisitions. Remember that a memorial ceremony does not need to be held for only newly obtained donors; donors or legacy tissues (e.g., skeletal remains, stored organs, or fetuses) may be re‐honored annually as part of a larger ceremony closing out the year or course.

##### Speakers and performers

Ceremonies provide an opportunity for the students, faculty, and donors' families to reflect upon the tremendous gift that is being able to learn anatomy from human bodies from any source (i.e., whether the remains are of body donors, unclaimed individuals, or of unknown provenance from legacy collections). Reflections can be in the form of speaking (i.e., reading a speech, poem, religious passage, etc.) or performing a song or dance. Speakers may include religious leaders, students, faculty, and/or family members of the deceased. Performers may include faculty, students, or individuals from music or dance departments not related to the anatomy course.

Common elements of a ceremony include opening remarks, faculty and/or student reflections on the gift of donation, musical selections or readings, a tribute to the donors (e.g., reading of the names or lighting of a candle), and closing remarks. Surveying students during the planning phase provides organizers an opportunity to determine how students desire to contribute to the ceremony. For example, students may wish to utilize their public speaking talents, musical abilities, graphic design abilities (e.g., creating the invitation, program, or a slide show), and/or displaying artwork. When inviting students to speak, be sure to be mindful of students' needs. For example, students who have recently lost a loved one or have known someone who has donated their body to science may be less comfortable speaking, or may be especially eager to speak.

##### Guests/attendees

While student presentations or performances at the ceremony should remain voluntary, attendance by students who directly worked with the anatomy donors in their course(s) should be strongly encouraged. In this way, all students are afforded the opportunity to show their appreciation and respect, and families (if in attendance) can realize the scope of the impact their loved one has made on future clinicians and professionals. Some programs/institutions may choose to make attendance mandatory, but this is a highly individual decision. Students who do not attend may be offered or required to complete an alternative method to pay their respects, such as a written reflection assignment or a thoughtful “thank you” token for families. If the ceremony is to take place outside of the regularly scheduled class time and student attendance is made mandatory, then care must be taken to select a date and time where all students are able to attend.

What other parties may be invited to your ceremony depends on the size and scope of your ceremony as well as on the location of the event. If your ceremony or honoring practice is to occur in the anatomy laboratory with the body donors, then only the faculty and students involved in the course are appropriate attendees. However, if your ceremony occurs outside of the laboratory, you may invite other faculty and students from across the campus, donor families, student families, or even individuals from outside the campus community to attend. Invitations could also be extended to local news media or the university media relations staff as a means to highlight and publicize the respectful actions of the institution/program or to advertise the donor program to the community. Approval from administrators may be required before extending the invitation to the larger community, especially to the local media. Keep in mind the seating capacity of your event location prior to sending invitations to make sure that your space can accommodate all whom you invite. Including a method for invitees to RSVP may assist in keeping track of potential attendee numbers.

Offering a livestream option allows you to include more attendees regardless of space limitations. A QR code or hyperlink to the livestream can be included on the invitation. Offering a livestream involves coordination with your institution's Information Technology (IT) department prior to the event to make sure that your location has the appropriate technology to offer this option and that all parts of the ceremony can be in the video and included with quality sound. Your institution may want to ensure that security settings for the stream are HIPAA‐compliant to protect family and donor privacy.

If you are inviting a large number of individuals not involved in your institution (e.g., donor families, student families, or the larger community), you need to consider parking or transportation accommodations as well as building access if doors to the building require key card or ID access. Speak with institution administrators as well as campus police to make all parties aware of the additional people on campus.

When deciding who to invite, be sure to follow the guidelines of your body donation program, which includes considerations about the privacy of the donors. Some organizations allow full names and personal information to be publicly shared; others consider all identifying information to be private. The desire for personal ceremonies and for open ceremonies may conflict, so compromise may be needed in the planning stages.

#### Location

##### Location of the ceremony

The size and timing of the ceremony influence the location of the event. Ceremonies may be held on‐campus or off‐site. Ceremonies for small groups of individuals (e.g., an anatomy course) may be held in the classroom or laboratory. Larger ceremonies may be held in an auditorium, campus chapel, off‐site religious space, or at an outdoor athletic field. Auditorium and outdoor venues may accommodate multiple classes, faculty, additional members of the campus community, and/or donor families.

Keep in mind what resources the space comes with or could handle, which may be finite and impose limitations on what your ceremony may include. If there is not an audio‐visual system in place in the room, for example, asking students to set up a slideshow would be unrealistic. Other important considerations for the location include accessibility, integrated technology, lighting, furniture and seating (and potential for rearranging seating), if food and beverages are permitted, and general comfort. Especially be mindful of legalistic and practical considerations such as ADA accessibility, fire code limitations, etc.

Depending on the size of the ceremony, planning committees may need to collaborate with other departments on campus to utilize space, such as larger classroom spaces, theater spaces, etc. Some institutions may choose to establish specific memorial spaces on campus (see *Institutional Markers of Respect (Memorials)*) and, in such cases, hold their ceremony at these locations. It is important to work closely with the individuals overseeing these areas to ensure they are available, have the proper technology support, and are presentable for the ceremony.

##### Inclusivity of the location

The ceremony should honor the beliefs and values of all participants and should not exclude individuals who wish to attend. Two major considerations when selecting an inclusive location for a ceremony are religious or cultural inclusivity and physical inclusivity. If a religious space, such as a chapel, is chosen as your location, attendees who do not follow that religion may not feel comfortable. Additionally, the donors' faiths are typically unknown, so honoring them by only a faith that they do not support could be disrespectful to them. However, a religious space can still be utilized for a ceremony since it may be the place with the most respectful atmosphere on campus. To keep a ceremony held in a religious space inclusive, care should be taken to make the rest of the ceremony components as interfaith as possible.

Physical inclusivity involves making sure that those with physical disabilities can enter the building and the room easily and that they have an acceptable place to sit. For example, if the location has unmovable seating, like pews, make sure that there is space for wheelchair seating.

#### Beneficial collaborations and partnerships

Planning memorial ceremonies involves collaboration among many people with various backgrounds. Cross‐departmental collaboration and community partnerships can be valuable during the planning stage and implementation of the ceremony. Incorporating diverse perspectives enriches the ceremony, offers educational opportunities about the significance of body donation, and helps develop empathy, gratitude, and compassion.

##### Body donation program

During the ceremony planning stages, the body donation program should be consulted to determine if there are any guidelines or regulations to consider, such as whether or not a donor's full name can be presented at the ceremony or whether the service can be live‐streamed or mentioned in media publications. Many donor programs hold an annual memorial ceremony and may be able to share sample programs and examples for your consideration. The ADS Committee can also assist with providing sample programs and examples, along with other planning resources as needed.

##### Institutional offices

During the planning stages, various campus entities, such as administration, facilities, security, and all relevant departments, should be apprised of the planning procedures. Additionally, as discussed further in the *Fundraising* section, collaborating with the institution's advancement/fundraising department may help secure funds for the ceremony costs. Campus Ministry or an institution's theology program may assist in developing interfaith ceremonies or aligning with a particular religious belief. Both entities can also help align programming with the institutional mission and core values.

##### Support services

The ceremony may emotionally affect those attending,^22^ so support services should be considered for input. The institution's campus counseling and mental health services may be consulted to discuss how to support the students' or other participants' emotional well‐being. This support may include campus counseling being present at the ceremony. They may remain afterward as a resource, or their contact information may be provided for those needing additional support.

##### Humanities interdisciplinary curriculum

The inclusion of the Humanities or the Fine Arts departments in the ceremony's planning not only introduces a variety of perspectives on ways to pay tribute to donors, but also provides the opportunity to develop an interdisciplinary curriculum that expands the educational impact of the donor's gift. Fine Arts departments, such as music, art, dance, and performing arts, can assist in contributing to the ceremony by providing guidance in planning or inclusion in the ceremony through music performances, readings, and artistic works. If the institution has Art Therapy or Music Therapy programs, incorporating these programs may help incorporate therapeutic methods for individuals to work through emotions. The institution's graphic design, marketing, or communication programs can assist in developing programs, memorial videos, and marketing materials.

Humanities engagement could also be useful in planning a ceremony around a broader theme, and in guiding planners in their program choices. For example, if the theme of a memorial ceremony is finding unity through brokenness, the Humanities department may suggest employing the ancient Japanese art form of *Kintsugi* (mending of broken pottery with gold) to visually symbolize this theme.

##### Community partnerships

Entities outside the institution may also have beneficial input or contributions to the ceremony. Considering the tone of your ceremony, local religious congregations may offer planning support and be a potential location to hold the ceremony. Area funeral directors hold both religious and secular services and may be able to provide selections for readings and music for the ceremony. Partnerships with local businesses, such as florists, may offer additional support in providing materials and decorations for the ceremony.

## COMPONENTS OF A CEREMONY

### Invitations and programs

Invitations provide the basic ceremony information for intended guests so that they can plan to attend. Invitations can be a printed page/flyer mailed to invitees and/or posted in locales across campus, or it can be a digital file emailed to invitees or posted on a website. If the details beyond the date and time of the ceremony are not confirmed, a Save‐the‐Date card or file can be sent out earlier to notify invitees so that they can plan to attend regardless of the location or theme of the ceremony.

Programs are documents, typically provided at the ceremony, that outline the order of events of the ceremony and list the names of the individuals speaking or performing. Similarly, a program can be a printed document, or it can be digital if your program/institution prefers a focus on sustainability. If the program is a digital file, you can place a QR code at the entrance to the ceremony location so that attendees can scan it to gain access to the file to follow along on their devices.

#### Responsibility for design

The design of invitations and programs for a donor memorial ceremony can be a collaborative effort between students and faculty. If any students have an interest in graphic design, communications, or the arts, the development of these documents provides them a chance to showcase their skills. Faculty advisors or administrative staff may provide guidance and support, ensuring that the designs align with the institution's standards and the respectful nature of the event, and that the documents are free from errors.

#### Design process

The design process should focus on creating a dignified and respectful presentation of the information. The printed or digital materials will be a lasting documentation of the ceremony, so care should be taken in their design and proofreading.

##### Concept development

Gather input from the planning committee or class on themes, colors, and elements that reflect the solemn and respectful tone of the ceremony. Consider incorporating elements that symbolize gratitude, reverence, and the educational journey. The content of invitations and programs should be carefully curated to respect the donors and their families while providing necessary information to attendees. As with all components of the ceremony, consider inclusivity (especially in the case of religious imagery) when designing the invitation and program.

##### Design elements

Decide on the amount and type of information to include, balancing between detailed and concise content. For all printed or distributable materials, remember to be consistent and appropriate in the use of religious imagery, pronoun choices, etc.

##### Invitations

Use a formal and elegant layout, incorporating the institution's branding subtly. Invitations should include the following minimum information:

- Title and host of the event: Include the name of the ceremony and who is hosting the event (for example, the “Physician Assistant Class of 2027 invites you to…”)
- Event details: Date, time, location, and dress code (if applicable)
- Purpose of the event: A brief statement about the memorial ceremony's significance
- RSVP information: Contact details for confirming attendance or a link/QR code to a form to collect RSVP information
- Livestream information (if including): A link or QR code for a livestream of the event.

##### Programs

Ensure the program is easy to read, with a clear structure and sections for each part of the ceremony. Include headers and other markers to organize content effectively. The program may be a full unfolded single page (single‐ or double‐sided), multiple unfolded pages stapled together, a page(s) folded to form a booklet, or a single page double‐folded to form a brochure. Programs have more variability in content than invitations, but they may include the following information:

- Title of the event, date, and an image or symbol representing gratitude and remembrance
- Welcome Message: A brief note from the dean, faculty, or a student representative expressing appreciation for the donors and the importance of the event.
- Event Agenda: Detailed schedule of the ceremony, including speakers, musical performances, and tribute activities.
- Donor Recognition: Depending on the consent given by donor families, it may be appropriate to include names, affiliations, and/or brief biographies of the donors. Some programs may feature photos of donors during their lives and may highlight their contributions and backgrounds to honor their legacies.
- Memorial artwork and writings created by students can be included within the program for later reflection.
- Acknowledgments: Thank you notes to donors, their families, the donation program, and all participants involved in organizing the event
- Support Resources: Information on counseling services or support groups for attendees who may need emotional support after the ceremony.

#### Review and approval

Obtain final approval from faculty advisors or administrative staff to ensure appropriateness and adherence to institutional guidelines. If students are in charge of the design, consider this approval process, the possibility of making changes, and turnaround time for printing when setting a deadline for them to submit a draft.

By thoughtfully designing the invitations and programs, the planning committee can create materials that not only inform but also honor the donors and convey the profound gratitude of the medical community.

### Refreshments

A simple reception with coffee, appetizers, and other refreshments, or a larger meal served before or after a donor memorial ceremony, can provide students, faculty, and other attendees an opportunity to share fellowship with donor families if they are invited, to reflect on the donors' life and educational impact, and to provide comfort to all attendees. Larger meals can be provided by local catering or a student‐organized potluck.

Potlucks, where students or attendees prepare and bring food and drinks to share, allow students and faculty to honor donors by showing their appreciation in a separate, yet equally meaningful way. If you choose to host a potluck, consider nominating student representatives to help ensure that you plan enough dishes to accommodate all of the attendees. Consider providing a variety of food and drink, and the timing of their offering, to accommodate many common nutritional and cultural needs (e.g., common allergies, low‐carbohydrate or similar for those with diabetes, sabbath, etc.). Additionally, remember to either assign individuals to bring non‐food items such as plates, cups, cutlery, napkins, and drinks, or otherwise arrange for these items to be present during the reception. As with all imagery involved in the ceremony, selection of these items should match the tone of the event. Providing and monitoring a sign‐up sheet for potluck items (food, drink, and non‐food) can be a task of the planning committee to ensure that there are enough items for all attendees.

If you plan to include a reception with your ceremony, you will likely need to get administrative approval to have food and beverage in the planned location. Additionally, your institution may have particular catering contracts that you will have to abide by, so consulting with administration early in the planning phase will ensure that you are following the proper channels.

### Readings

Readings tend to be the bulk of typical donor memorial ceremonies and can provide faculty, students, or donor family members an opportunity to read something meaningful to them to honor the donors. Readings can be a speech or poem written by the reader, a published poem or other text, or a religious passage. The readings do not necessarily need to be specific to body donation or the benefits of the donation gift, but only need to have meaning to the reader, which can be included as a preface or conclusion to their speech. Therefore, there are endless possibilities for ceremony readings, so it is helpful to provide volunteering speakers with a list of example readings to help them generate ideas when identifying a reading that is meaningful to them (Table 1). You could also reach out to local funeral homes for additional recommendations for passages that inspire comfort or gratitude.

**TABLE 1:** List of reading ideas relevant for body donor memorial ceremonies.

**Secular Reading Ideas**
*Knowing You* ^64^
*Anatomy Teacher* ^65^
*The Calling*.^50^
**Christian Reading Ideas**
Psalm 139
Philippians 4:5‐9
Corinthians 12:12‐27
**Jewish Reading Ideas**
Mourner's Kaddish (only if at least 10 Jewish individuals are present)
*We Remember Them* ^66^
*Prayer in Place of Mourner's Kaddish* ^67^
**Hindu Reading Ideas**
*Bhagavad Gita* verse 4.34
*Tvameva Mata*
*Sarvetra Sukhina Santu*

If religious readings are to be included in the ceremony, it is best to include readings that represent various faiths to promote inclusivity. Furthermore, it may be helpful when choosing readings to select passages that emphasize themes that are common to multiple faiths.^68^, ^69^ For example, the Abrahamic religions (i.e., Judaism, Christianity, and Islam) share belief in one sole transcendent God, a final judgment, and an eternal afterlife. Additionally, core themes revolve around justice, charity, comfort for the poor and the meek, the importance of prayer and fasting, and righteous living. The Dharmic religions (i.e., Hinduism, Buddhism, Jainism, and Sikhism) share themes revolving around the cycles of creation and destruction, the importance of self‐reflection and spiritual growth, the ethic of reciprocity, and the idea that present actions have consequences that impact the present as well as the future, including consequences for rebirth.^70^, ^71^ Despite the readily apparent differences between the religions, universal ethical and moral frameworks are still shared—values such as compassion, truthfulness, justice, honesty, non‐violence, love for one's neighbor, duty, acknowledgement of a higher reality and the humility that should come with that, and belief that something happens after we die.^72^, ^73^, ^74^, ^75^

When selecting readings from specific faiths, care must be taken to ensure that the religious readings conform to any regulations or conditions that may be a part of that faith. Although Christian prayers generally have no restrictions on who can recite them, some denominations and cultures may have their own practices or traditions surrounding specific ones and should be consulted individually. Reading passages from the Torah or the Mourner's Kaddish, a Jewish passage recited in memory of the dead during the mourning period after death, cannot be read unless a *minyan* (a quorum of at least 10 Jewish individuals) is present.^76^ Reading these religious passages without the presence of a *minyan* would be considered disrespectful to any Jewish attendees.^14^ However, there are other readings that reflect the Jewish faith that are not formal religious passages, so they can be read without the presence of a *minyan*. One example is *We Remember Them*, which was written by two rabbis.^66^ This passage is particularly useful for encouraging attendee participation in the ceremony since each line of the poem ends with “we remember them,” which the attendees can recite along with the reader. Another example is the *Prayer in Place of Mourner's Kaddish*, also written by two rabbis to assist gatherings of mourners who do not meet the *minyan* requirement.^67^

Hinduism can be incorporated into the ceremony by having students or faculty recite a Hindu prayer or mantra. The mantra can be recited in Sanskrit and an English translation or description of the meaningfulness of the mantra included in the speech or in the ceremony program. For example, a Seton Hill University Class of 2021 Physician Assistant student read the mantra *Tvameva Mata* in Sanskrit and provided the following description in the ceremony program: “This prayer recognizes that the Gods leave an impact on us in so many different ways and in our situation, we believe that the donors left a godly impact on our lives by choosing to donate their body to science in order to help us become better providers one day.” Another student from the same class read the mantra *Sarvetra Sukhina Santu* in Sanskrit and offered this description: “This prayer is about blessing souls with eternal happiness. We are expressing our gratitude to the donors by praying that they do not face any negative situations in the afterlife nor experience any sort of sadness. With this prayer, we pray that these donors know that they are appreciated.”

Including Islam into the ceremony can be as simple as referencing the Prophet Muhammad, who said “The best of people are those who are most beneficial to others” (Al‐ Mu'jam al‐ Awsat; 5937), which can then be used to describe the altruistic gift of the body donors in a speech. Alternatively, a passage from the Quran *c*an be read, or a formal du'a (prayer) can be recited, which can be in Arabic or translated into English. Figure 1 presents a du'a written by Abdul Mawgoud Dardery, PhD, to specifically thank and honor human body donors. Hadie and colleagues provide a detailed description of Islamic views on body donation, human dissection, and honoring body donors at ceremonies, which may provide further ideas.^77^

**FIGURE 1:**
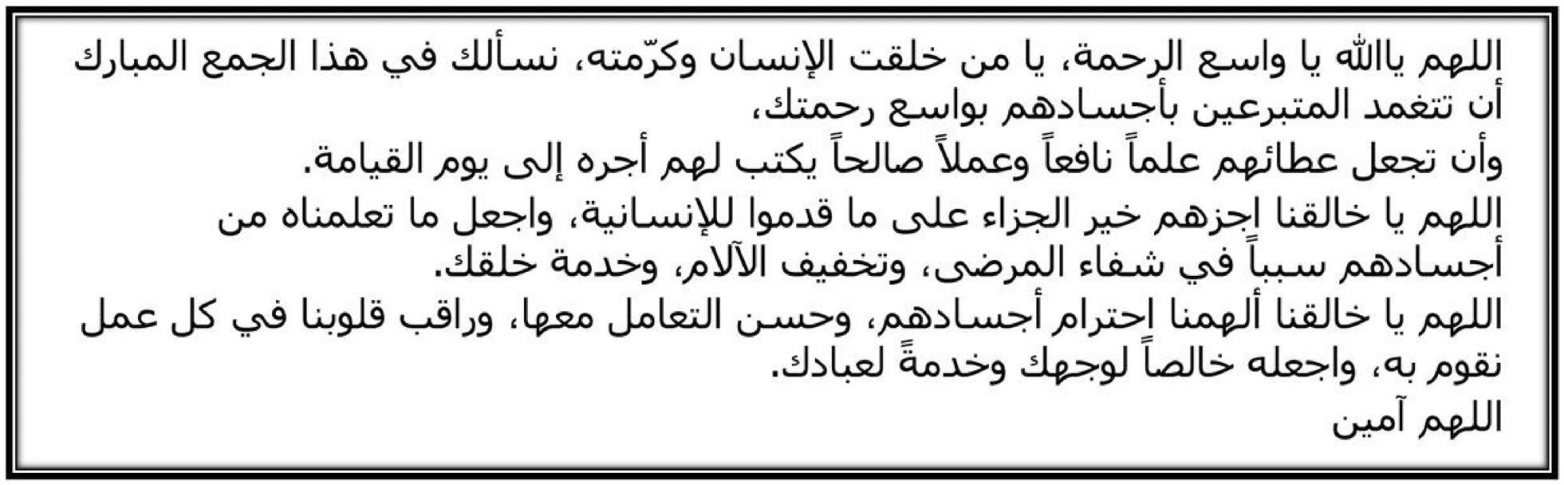
Du'a for Human Body Donors By Abdul Mawgoud Dardery, PhD. Translation: “O Allah, Most Merciful and Most Generous, You who created humankind and honored them, we ask You in this blessed gathering to envelop those who donated their bodies with Your vast mercy, and to make their gift a source of beneficial knowledge and a righteous deed that continues to earn them reward until the Day of Judgment. O God the Creator, grant them the best of rewards for what they offered to humanity. Make what we have learned from their bodies a means for healing the sick, easing suffering, and serving Your creation. O God the Creator, inspire us to treat their bodies with respect, to handle them with care, and to keep our intentions sincere in every action we take. Make our work purely for Your sake and in service to Your servants. Ameen.”

Any passage has the potential to be meaningful or moving to a student if it speaks to them, irrespective of faith background or cultural tradition. This can be as much due to what the student's life story has been or what the student may privately be living through in that moment as much as anything else. And so, when incorporating student‐chosen readings, it may not be worth scrutinizing the degree to which the passage addresses human body donation or even death directly, as long as it is otherwise contextually appropriate. However, if the reading does address dissection or what specifically happens to the donors, ensure that the descriptions are suitable for all audiences and sensitivity levels and that the reading may not be viewed as offensive to those present or to the donors.

### Music

Music is a powerful component of a ceremony that can evoke emotions as well as provide an opportunity for students' artistic talents to shine. Music can be incorporated prior to the commencement of the ceremony (i.e., while attendees are entering the venue), during the ceremony events, and at the conclusion of the ceremony (i.e., while attendees are mingling and exiting). Playing music, either from a prerecorded form or as a live performance, as attendees enter the venue can set the tone for the ceremony and reduce the awkward silence that can occur before the event begins. Music can also be played or performed as part of the ceremony, as a reflective performance within the program, as an interlude between speeches, or during otherwise silent events within the program (e.g., the “Giving of the Rose” ceremony described below). Live performances (singing or instrumental) can be completed by student volunteers or by outsourcing to the music department or outside community. If student volunteers are requested to perform, ensure that the request is optional and that performers do not feel pressured by faculty or other students since preparing for a performance can be stressful.

Music is another component of the ceremony where the amount of religion included should be considered, as songs performed could be secular, religious, or a mix of both depending on the factors listed in the *Religious Involvement* section. It may be helpful to provide volunteering performers with a list of example music to help them generate ideas when identifying a song to perform that is meaningful to them (Table 2).

**TABLE 2:** List of music ideas relevant for body donor memorial ceremonies.

**Secular Music Ideas**
*For Good*, from the musical, *Wicked*
*Teach Your Children*, by Crosby, Stills, and Nash
*I'll Remember You*, by Bob Dylan or Thea Gilmore
*My Old Friend*, by Tim McGraw
*One Sweet Day*, by Boyz II Men and Mariah Carey
*Ripple*, by The Grateful Dead
*In My Life*, by The Beatles
*Tears in Heaven*, by Eric Clapton
*The Rose*, by Bette Midler
*Wind Beneath My Wings*, by Bette Midler
*Let it Be*, by The Beatles
*Stop Crying your Heart Out*, by Oasis
*Imagine*, by John Lennon
*And so it Goes*, by Billy Joel
**Religious Music Ideas**
*Hymn of Promise*, by Nancy Allen
*Hallelujah*, by Leonard Cohen
*It Is Well With My Soul*, by Horatio Spafford
*Jesus You Have Come Down To The Lakeshore*
*Great Is Thy Faithfulness*, by Thomas Chisholm (composer: William M Runyan)
*His Eye Is On The Sparrow*, by Civilla D. Martin
*I'll Fly Away*, by Albert Brumley
*I Can Only Imagine*, by Mercy Me
**Interfaith/“Spiritual” Music Ideas**
*Danny Boy*, by Joseph Flummerfelt
*What a Wonderful World*, by Louis Armstrong
*Time to Say Goodbye*, by Andrea Bocelli and Sarah Brightman
*You Walk with Me*, by David Yazbek
*Somewhere Over the Rainbow*, by Israel Kamakawiwoʻole
**Instrumental Music Ideas**
*At Peace*, by Thomas Smith (piano & viola)
*Give Thanks*, by Phillip Keveren and David Angell (piano & violin)
*After the Rain*, by Kevin Kern (piano)
*Where Paths Meet*, by Kevin Kern (piano)
*Journey*, by Jim Brickman (piano)
*Bittersweet*, by Jim Brickman (piano/piano & strings)
Clair de Lune, by Claude Debussy

Students or faculty may also write their own music to perform to make the piece even more meaningful and relevant to the ceremony. For example, an anatomy professor at City University of New York wrote a song specific to honoring body donors to perform at their memorial ceremony.^52^ ^(p76)^

### Ways to include donor families

If possible, including families or loved ones in the memorial can serve as closure or the end of a liminal period, as the donor has now completed their time with the institution and has been or will soon be returned to the family. Student and faculty support and participation in the memorial ceremony can have a profound impact on donor families and loved ones.^17^ At the memorial, faculty or students may share their experiences that emphasize the importance of donors in their teaching and learning, allowing the families to hear from those who benefited from their loved one's gift, providing them a glimpse of the direct impact their loved ones had on so many individuals. This demonstration of love and appreciation for the donor and their gift can leave a lasting positive impact on all those in attendance. Additionally, faculty and students might write notes of gratitude, offer flowers, or give other tokens of appreciation to the families, which can serve as tangible symbols of the donor's impact. Reading or displaying the donors' names (first only or full, depending on donor program regulations or donation form consent) can also create a meaningful tribute.

Some donors or families may want specific personal or religious effects (photos, rosary beads, quilts, jewelry, etc.) to be included in the ceremony or the cremation. Donors who choose to give to a particular program or institution may wish to be remembered with items of personal significance, such as university memorabilia. Displaying these items during the memorial or including them in the cremation not only personalizes the donation process but also honors the wishes of the donor and their family.

After the donor has completed their time at the institution, their cremated remains may be returned to the family or other loved ones. Some families may choose not to accept the remains, so it is essential to have a location for their interment, such as at a local cemetery, in which case a memorial to the specific donor (temporary or permanent) may be placed. In this case, a small ceremony may take place at the interment site in which family members and students could attend. In cases where families wish to have their loved one returned, cremated remains can be hand‐delivered or picked up at a specific location. This interaction again allows for a formal end to the donation process.

### Tokens of remembrance and activities to include in the ceremony

- **Cards:** Cards or letters for the donors and/or their families can be made by several students before the ceremony and then signed at the event. Letter writing also provides students a reflective outlet^41^ and a means to provide a gift to the donor or their family in thanks for the donation of their body.
- **Artwork:** Inviting artistic students to contribute paintings (see Figure 2), drawings, framed poetry, or even sculpture is a great way to honor human body donors and to encourage participation. These items can be displayed at the ceremony or reception. The entire class could also participate in an art‐making workshop guided by an Art Therapy department as an additional method to help students cope with the dissection process.^78^ In this case, the artwork from the entire class can be displayed during the ceremony as tributes to the donors, making the event more personal to the students.
- **Candles:** Candles can be displayed to represent each donor and may be lit by students or family members. An explanation of the candle memorial can be given by a ceremony speaker or included in the paper program. The candles can be lit prior to the start of the ceremony to contribute to the ambiance as attendees enter the venue and to avoid any lighting issues (e.g., a lighter malfunctioning) or awkward silences during the ceremony. Alternatively, the lighting of the candles can be completed as part of the ceremony service. If the latter method is utilized, test the lighter beforehand and have a replacement easily accessible if needed.
- **Flowers:** Students can pick or purchase flowers and arrange them for the ceremony. To make this more affordable, consider soliciting your institution directly for funds, or reach out to establish a partnership with a local florist or flower farm. Roses are a common flower to depict love and gratitude that are easily obtained and may be dyed various colors. Each rose color has a different meaning, so you could make the flower selection even more personal for the students by allowing them to select a rose color to represent all of the donors or for each individual donor.
- **Giving of the Rose ceremony:** Instead of displaying an arrangement of flowers at the ceremony, active participation can be included by having each student, faculty member, or even all attendees place a single flower (type and color selected by the students or planning committee) into a vase during the ceremony. Depending on the number of donors you are honoring, you could have a vase to represent each donor, and only the students assigned to the particular donor would place their flower in that vase. In this case, each dissection group could select a rose color that is meaningful to them in honoring their particular donor. This ceremony element provides a means for each student to physically give something back to the donors without them having to speak or perform during the ceremony, allowing every student an opportunity to participate in the ceremony and can make the ceremony more individualized and meaningful to the students.
- **Reflection essays, discussions, or poems:** Students can provide a reflection on their experiences working with donors, how it impacted their education, and express appreciation to the donors and their families. Reflections can also be written as a letter to the donor or the donor's family, or even as a poem. Reflections can be selected to represent each participating program or group of students. Due to the benefits of reflection, completion of them can be encouraged by integrating them into the course as an assignment.
- **Video of thanks created by students:** Students can create a video of classmates expressing their thanks to the donors to play during the ceremony or to house on the university/program website for a more permanent memorial (see the *Institutional Markers of Respect* section). The students then have a means to personally thank the donors without having to speak or perform at the ceremony.

**FIGURE 2:**
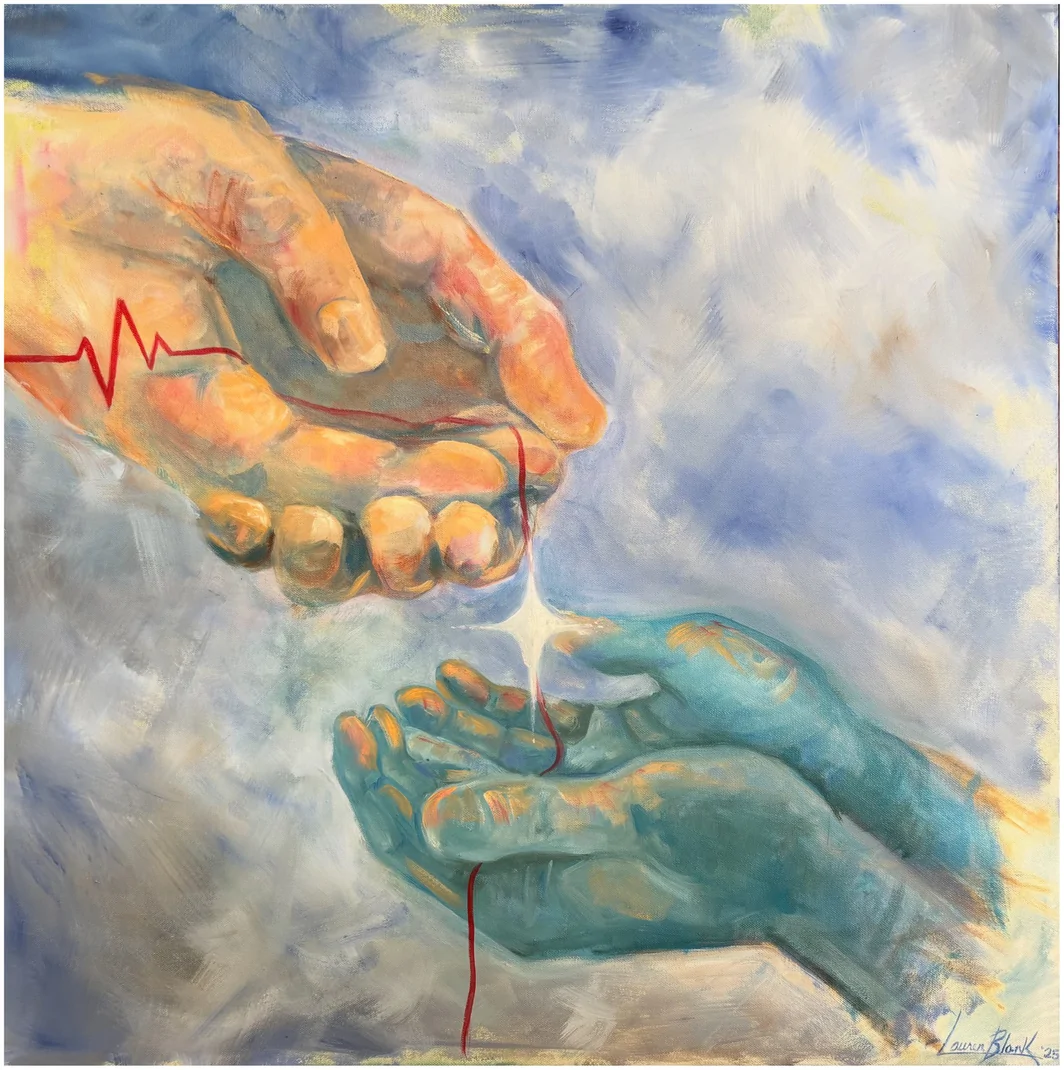
Painting by Lauren Blank, a Lipscomb University student, for her donor memorial ceremony. She designed this thinking about the flow of blood in the donor transitioning with the “spark” to the gloved student's hand.

#### Honoring military veterans

Additional elements can be added to the ceremony to honor donors who have served in the military. These individuals served in life and then donated their body to continue serving after death, so they deserve additional acts of gratitude and honor. Tributes to veterans could include displaying a folded flag or flag symbols in the ceremony (e.g., a small flag in a vase of flowers or flags represented in student artwork to honor the donors), playing of “Taps” at some point in the ceremony, or having a current military person or veteran do a formal salute in honor of the donor(s).

## OTHER END‐OF‐COURSE HONORING PRACTICES

Ceremonies to honor body donors do not need to be elaborate or hugely attended to be meaningful, nor does the ceremony need to be confined to one grand final gesture at the end of the semester. Smaller gatherings and thoughtful inclusion of intimate moments can be every bit as impactful to your students.

### Devotion of a class day

The memorial ceremony, particularly if attended by only anatomy faculty and students, may be held in the anatomy laboratory or anatomy classroom itself during a dedicated class time. By featuring this time and date on the syllabus from the beginning of the semester, students can mentally and emotionally prepare for the event. A small class‐day ceremony can provide students with a similar sense of gratitude for their community and a specific shared experience with their donor, similar to a funeral. Some students may feel freer sharing words, art, music, or their emotions with a smaller group of people who are familiar to them and have shared the same experiences in the laboratory.

### Moment of silence or emotional reflection

Offering students a moment of silence for emotional reflection can be a simple but impactful way for students to pause and remember the donor's gift. This moment or reflection time can occur at the beginning, during, or at the end of a class day or at the end of the semester. It can also be a gesture unto itself or incorporated into a larger tribute practice.

Students may be provided the opportunity to share feelings and thoughts that arise afterward, or the moment of silence may be framed in such a way that students feel guided to say their final thanks and goodbyes silently during that moment. Depending on the instructor's preference and the information available to them, some donor information (e.g., their name) may be revealed immediately before or after a moment of silence. In lieu of an informal sharing moment after the moment of silence, the instructor may prefer to hold a separate, more directed discussion session with students.

For more privately shared reflections, students may be encouraged or required for a grade to write a reflection essay describing their experience with their anatomical donor. This formal written reflection can be as complex or unstructured as you find appropriate for your course.

### Offering flowers or other “gifts” for donors

Students may benefit from the opportunity to provide a gift of some kind to the donor at the end of the semester to physically thank them for the gift of their donation. This practice is reflected in many cultures and religions in funerary practices for a variety of reasons. This gesture can be small and individual to each student, or it can be a single gift agreed upon by the group. Note that this item must be something that can be cremated safely with the donor, and the family may need to be consulted prior to their loved one being cremated with this item.

### Blessing of the donors

A blessing of the donors can occur in lieu of or in addition to a memorial ceremony. This event involves formally blessing the donors once dissections have been completed as a means to wish them well in their next phase (i.e., cremation and internment or return to their families). Even though a blessing is typically associated with religion, it can be conducted in an interfaith manner in order to be inclusive to all faiths. If religious components are included, those elements can be made optional, or components from multiple faiths can be incorporated into the blessing service. The blessing could be led by a religious leader who is willing to keep the service interfaith, a faculty member, or even a student. Whereas a memorial ceremony acts as a ‘thank you’ to the donors, a blessing of the donors acts as a final farewell to the donors.

This final farewell to the donors could be as simple as described by offering them an interfaith blessing, a prayer, a moment of silence, or placing a flower on them prior to leaving the lab for the last time. Conversely, a more formal farewell ceremony that includes the donors' families could be organized. For example, at the conclusion of dissections in Taiwan, students dress the donors in white garb, place them in coffins, and accompany the transport of the coffins down the street lined with family members and other invited guests to the crematorium.^40^, ^41^ This public funeral service allows anyone in the community to attend and pay their final respects to those who have given the gift of their bodies for education.^42^

### Tokens and rituals of respect/reverence

Rituals of respect for the donor can be intimate and deeply meaningful for students and can provide students with an activity to do that feels actively reverent. Activities in particular that mirror those employed by some faiths (e.g., cleansing of the donor prior to first dissection and/or after the last dissection, shrouding in fresh linens, etc.) can be emotionally evocative and even among religious students may convey an added sense of tradition and solemnity to what can otherwise be a disorienting experience. Some activities, like replacing organs or tissues removed from the body during the semester to the donor or neatly restitching together skin flaps, may convey a sense of restoration and “fixing” to students who felt uneasy about the dismemberment of their donor.

Small daily routine tasks can also be impactful and allow students to demonstrate gratitude and remind them daily of the gift of that donor. Students may be asked to say “hello” when they first reveal their donor for the day, or “thank you” before putting away their donor for the day. Students may take a brief moment of silence or have a brief conversation before dissecting to ensure that the donor is treated in the way they might hypothetically approve of best (e.g., ensuring incisions are made around and not through tattoos where possible, considering how to dissect in a tidy way, etc.). Activities such as spraying the donor and daily re‐shrouding can be framed as ritualistic caregiving and allow students to transition from the anatomy lab to the rest of their day with greater closure. Keeping the body as intact as possible during and after dissections provides another opportunity for students to show caregiving, empathy, and respect.^58^, ^79^ Students should be instructed to replace all organs and skin at the end of each laboratory session and also at the conclusion of the course as a final act of respect. They could even suture the dissection cuts at the end of the course to ensure the donor is returned as complete as possible, as is the practice in Taiwan.^38^, ^39^, ^40^, ^41^, ^42^ Keeping the donor intact during the semester may help maintain their humanity during the dissection process^14^ and will help ensure that as much of the body as possible is interred or returned to the family when dissections are complete.

## INSTITUTIONAL MARKERS OF RESPECT (MEMORIALS)

To continue to honor donors for their gift to the education of students, institutions can place permanent tribute markers on campus, on university websites, at cemeteries/memorial gardens, etc., in addition to hosting memorial ceremonies. These memorials are varied in presentation but have the overall goal of providing a permanent physical space to remember human body donors and their gift to education.

As with ceremonies, memorials should be inclusive of all faiths and cultures as the religions of the donors are not always known. Honoring a donor with a permanent marker with religious iconography not in line with their own faith could be insulting and upsetting to their family members. Grachan et al.^47^ discuss the importance of considering the donors' varied faiths in designing memorials.

### Public memorial markers

Memorial markers can be placed in any publicly accessible area. These markers are most often found on educational campuses where human body donors donated their bodies. These can range from a simple dedicated plaque to elaborate sculptures and living memorials (e.g., tree planting). An example of a creative public monument to body donors meant for families and students to visit was an old marble dissection table fashioned into a monument by a medical school in the Netherlands.^80^

Some institutions allow for donors and/or donors' families to include details such as name, hometown, year of birth and death, etc. to more directly identify them in the tribute. If schools utilize outside donor organizations, they should coordinate with that organization to respect their policies.

### Entrance to the anatomy laboratory

Some institutions honor donors in the building they are placed for educational investigation with a dedicated plaque, tribute wall,^81^ or a book of remembrance. A book of remembrance is a record of donors' names put on display in the building where donors are located.

### Website tributes

Dedicated website tributes for donors and donor families can be found on many human body donor program websites. These sites can range from a dedicated statement, poem, or video. Videos are generally organized as an abbreviated memorial ceremony with song, poems, speeches, etc., or as an additional means for students to say a short ‘thank you’ to the donors. Memorial websites can provide a way for donors' family and friends to see the educational impact of their loved one's donation without travel or limitation of time (i.e., a ceremony offered only at a specific day/time/location).^82^ If a school wishes to create a memorial website, the maintenance of that website should be planned for. Broken links and navigation diminish the respectful presentation for which the site was created.

### Cemetery space

Dedicated cemetery space is a common way to not only honor donors with a public memorial, but also allows for the internment of donor cremated remains after return of the body. If donor cremated remains are not able to be spread or interred at cemeteries, messages of thanks, hope, etc., can be collected, burned, and those cremated remains spread at the dedicated monument.

### Dedicated garden or tree

As an alternative to a memorial statue, a dedicated tree, plant (such as a rose bush), and/or garden can give thanks to donors and their families while providing a peaceful space for family, friends, and students to visit. Dedication can be from the school broadly and represent all classes that worked with human anatomy donors generally, or individual donations can be given by each graduation year.

### Reflection bench

A dedicated bench in a garden, cemetery, or other peaceful environment can provide space for reflection and thanks to students and to donors' families.

## FUNDRAISING

Depending on the cost of the planned ceremony and/or memorial gift, it may be necessary to raise funds. Close collaboration with your administration is key to maximizing the amount of money your institution is willing to contribute. Your relationships and promotion will determine how much money you will have to independently source for the successful production of your ceremony. These discussions should be had as early as possible, and ideally before the source's budgeting deadline, to provide ample time to plan and execute additional fundraising activities, if necessary. The goal of fundraising should be only to raise the funds necessary to hold the event. Neither the ceremony event nor the body donors themselves should be utilized for commercialization or monetization purposes, as neither should be a source of income for the institution or program.

### Potential sources for funding

Note that in this section only, people who donate money may be referred to as “donors” or “financial donors”. Any mention of “anatomical donors” will be specified as such to distinguish the two entities. When discussing plans with your administration, you should use similarly clear terminology, as to them, “donor” will always mean “financial donor”.

Sources of funding can vary for this type of event/gift and may include:

- **Direct Support From Your Administration:** Your administration (department, school, or university) may be open to providing financial support for your ceremony. This support could include allocating a portion of existing departmental or institutional budgets, offering discretionary funds, or identifying specific grants or funding opportunities within the organization. To increase the likelihood of securing support, prepare a clear proposal outlining the purpose, budget requirements, and anticipated impact of the ceremony. Engaging with administrators early and demonstrating how the event aligns with institutional values or goals can also strengthen your case.
- **Allocation in Student Lab/Course Fees:** When determining fees associated with your course, it may be appropriate to include a line item for the ceremony. Typically, lab/course fees are calculated using a zero‐based budget, meaning the ceremony budget should be planned in advance and distributed across the number of students enrolled in your course(s).
- **Endowment Funds**: An institutional endowment fund is a dedicated account within the institution where monetary gifts are restricted to specific purposes, such as memorial ceremonies or monuments. Endowments may consist of contributions from multiple donors or a single individual and can be structured as one‐time or recurring gifts.
- **Community Fundraising:** Donations from your immediate community can be sought on an individual level, such as reaching out to friends and family, or through organized fundraising events. Local businesses might support your cause by hosting a fundraising day, where a portion of their proceeds is donated, or by setting up a dedicated donation box for contributions.

Solicitation of funds from outside sources should be done with care and still should come under advisement by your host institution. Your institution will typically have a budgeting or endowment office that can help with managing incoming funds for this (for legal and/or tax purposes).

- **Deposit location:** Under no circumstances should you deposit donated funds into personal accounts, even temporarily. An account should be identified or established within your institution as the sole destination for incoming funds from financial donors. Depending on your local, state, or federal laws, or institutional preference, donations may be limited to cash or check, while credit card donations are not allowed.
- **Tax information:** Depending on your local, state, and federal laws, the bequeathment of funds by outside parties may be tax‐exempt, but not always. Do not promise your donors that they can “write off the donation” on their taxes, or provide any similar incentivizing. By establishing your accounts through your institution, you can ensure that withholding of taxes, filing for tax exemptions for donors, or invoicing vendors on a tax‐exempt status is handled legally and appropriately.
- **Website Donation:** Aside from soliciting individuals directly, indirect vehicles for donation may be established (like setting up a link on your department's website) to reach a larger audience.

### Marketing and media

Properly marketing your donor memorialization event or an event to present a permanent memorial marker may provide your institution or program with desirable exposure, allow program alumni to express their gratitude towards the donors, and may even provide awareness about human body donation. These materials may include personal perspectives or student testimonials, interviews, photographs, or videos intended to be shown at or to promote your ceremony. Promotion of these events can be shared via social or printed media. Regardless of the method, before marketing your ceremony, it is necessary to first reach out to your body donor institution and your university administration to obtain guidelines that will ensure that you follow protocol and produce materials that are appropriate and ethical (and not sensationalizing) to the donors, their families, and the program.

Marketing strategies may include working with a team to design posters, writing a press release for your university's social media or website, and/or requesting coverage from the university or local newspaper. Your marketing department may have an online form where you can outline your needs; however, you may also want to meet with a representative in person to discuss resources and limitations. Regardless, materials intended for distribution to the general public should be released well in advance (approximately 4–6 weeks) to maximize potential for participation.

Bear in mind that marketing memorialization of anatomy donor programs may be construed by some as solicitous, hawkish, or in poor taste by its very nature. Marketing is not in, and of itself, a bad thing, but institutional involvement is crucial to avoid embarrassment or poor publicity.

## SUMMARY AND REFLECTION

Donor appreciation ceremonies and tributes play a vital role in allowing students, faculty, and staff to express gratitude to anatomical donors and their families, while also giving students an opportunity to reflect on their experiences with the donors. These tributes can take many forms, but the most important consideration is honoring the donors and their altruistic contribution to anatomy education. This text aims to share examples and practical details that may help others in planning these meaningful events. The Anatomical Donor Stewardship Committee is pleased to support anyone creating or refining their donor appreciation tributes and welcomes any questions or requests for guidance.

## AUTHOR CONTRIBUTIONS

**Bobbie J. Leeper:** Conceptualization; writing – original draft; writing – review and editing; supervision; project administration; investigation. **Rhiannon E. Robinson:** Conceptualization; investigation; writing – original draft; writing – review and editing. **Trisha Waldman:** Writing – original draft; writing – review and editing. **Nicole L. Geske:** Writing – original draft; writing – review and editing. **Jill Kirby:** Writing – original draft; writing – review and editing. **Danielle N. Edwards:** Conceptualization; writing – original draft; writing – review and editing. **Jacqueline Phillips:** Writing – original draft; writing – review and editing. **Kelsey Stevens:** Conceptualization; writing – review and editing; writing – original draft. **Lacy M. Cleveland:** Writing – review and editing; writing – original draft. **Burhan Gharaibeh:** Writing – original draft; writing – review and editing. **Jonathan J. Wisco:** Writing – original draft; writing – review and editing. **Leslie R. Sewell:** Writing – original draft; writing – review and editing. **Jeremy J. Grachan:** Writing – original draft; writing – review and editing; conceptualization; investigation; project administration; supervision. **J. P. Swigart:** Writing – original draft; writing – review and editing. **Anya Goldina:** Writing – original draft; writing – review and editing.

## Supporting information


**Appendix S1:** Supporting Information.

## Data Availability

Data sharing not applicable to this article as no datasets were generated or analysed during the current study.
