# Supplementary material for: The gift remembered: The Human Anatomy and Physiology Society (HAPS) Anatomical Donor Stewardship's ethical and practical handbook for anatomy donor tributes
Source: Anat Sci Educ. 2026 Jul 16;19(11):1802–22. doi: 10.1002/ase.70309 (PMC13736540; doi:10.1002/ase.70309)
Supplement: Supplementary file 1 [file ASE-19-1802-s001.docx]

**Tasks for Active Planning & Implementation of a Memorial Ceremony for Human Body Donors**

- Obtain all necessary permission from your institution re: if you can hold a ceremony and if so, what scope of ceremony may be
- Determine date & location for ceremony
  - Identify institutional points of concern
    - ADA accessibility
    - OSHA / fire code regulations
    - Parking needs / access to building
    - Liability & insurance
    - HIPAA & other privacy concerns
- Draft invitations - at minimum, save-the-date
  - Create guest list with roles (speaker, musician, guest, etc) and contact information
    - Create QR code for RSVPs, virtual programs, and/or for link to livestream (below)
  - Send min 4 weeks prior to event
  - Live-stream for non-physical attendees
    - Obtain permission and know tech parameters for livestreaming from your institution (e.g., encryption, HIPAA compliance, etc)
- Draft ceremony program (physically and strategically)
  - Design artwork for program
  - Plan timing of ceremony and consider transitions
    - List each step of the ceremony in an intuitive, natural-flowing way (speakers, readings, music, tribute activities, reflections, moments of silence, etc)
    - Assign an approximate amount of time each component should take and make speakers / presenters aware of that time
    - Consider how to move from one presentation type to another (e.g., introducing the next speaker, etc)
  - Create physical programs
    - Include time & date and personal information permissible / preferable by Institution
    - List acknowledgements (who helped create ceremony, acknowledge donors and families as well)
    - Assign someone to print (and determine payment for) physical programs
- Create participant / volunteer list
  - Speakers and performers
  - General organizer / project manager
  - Liaison with administration (permission, dates & location, safety / legal considerations, funding sources / fundraising, securing parking passes, etc)
  - Person in charge of…
    - Creating invitations & programs
    - Hosting / identifying host (professor, clergy, etc)
    - Creating marketing materials & advertising items
    - Ordering & arranging of flowers, decorations, and artwork
    - Ordering food
    - Initial setup / breakdown of site
      - Cleanup
      - Furniture
    - Managing candles / lighters
    - Distributing tokens of remembrance to guests
    - A/V equipment (identification, management during ceremony, including lighting)
    - Paperwork / treasurer (e.g., managing receipts & budget)
- Identify necessary AV equipment for room (e.g., sound system, microphone, cameras, etc)
- Select readings and assign readers (~5-6 readings max)
- Select music for ceremony
  - Playing as people enter / before ceremony
  - Played during different components of ceremony (e.g., Giving of the Rose component, to end moment of silence, etc)
  - Playing during reception or as people are leaving
  - Assign a person responsible for music (functionality, timing, volume, feedback, etc)
- Flowers and other decorations (e.g. vases, candles, etc.)
  - Color selection / color theme / flower theme (color, type of flower, season, etc)
  - Assign a purchaser and decorator for day-of
- Artwork
  - Identify artwork you would like to display and its source (e.g., storage, student-created, etc)
  - Identify what infrastructure is necessary to display art (e.g., easels, etc)
